# Gene Expression Signatures That Predict Outcome of Tamoxifen-Treated Estrogen Receptor-Positive, High-Risk, Primary Breast Cancer Patients: A DBCG Study

**DOI:** 10.1371/journal.pone.0054078

**Published:** 2013-01-16

**Authors:** Maria B. Lyng, Anne-Vibeke Lænkholm, Qihua Tan, Werner Vach, Karina H. Gravgaard, Ann Knoop, Henrik J. Ditzel

**Affiliations:** 1 Institute of Molecular Medicine, University of Southern Denmark, Odense, Denmark; 2 Department of Pathology, Odense University Hospital, Odense, Denmark; 3 Department of Pathology, Slagelse Hospital, Slagelse, Denmark; 4 Department of Clinical Genetics, Odense University Hospital, Odense, Denmark; 5 Institute of Public Health, University of Southern Denmark, Odense, Denmark; 6 Institute of Medical Biometry and Medical Informatics, University Medical Center Freiburg, Freiburg, Germany; 7 Department of Oncology, Odense University Hospital, Odense, Denmark; 8 Department of Oncology, Rigshospitalet, Copenhagen, Denmark; Massachusetts General Hospital, United States of America

## Abstract

**Background:**

Tamoxifen significantly improves outcome for estrogen receptor-positive (ER+) breast cancer, but the 15-year recurrence rate remains 30%. The aim of this study was to identify gene profiles that accurately predicted the outcome of ER+ breast cancer patients who received adjuvant Tamoxifen mono-therapy.

**Methodology/Principal Findings:**

Post-menopausal breast cancer patients diagnosed no later than 2002, being ER+ as defined by >1% IHC staining and having a frozen tumor sample with >50% tumor content were included. Tumor samples from 108 patients treated with adjuvant Tamoxifen were analyzed for the expression of 59 genes using quantitative-PCR. End-point was clinically verified recurrence to distant organs or ipsilateral breast. Gene profiles were identified using a model building procedure based on conditional logistic regression and leave-one-out cross-validation, followed by a non-parametric bootstrap (1000x re-sampling). The optimal profiles were further examined in 5 previously-reported datasets containing similar patient populations that were either treated with Tamoxifen or left untreated (n = 623). Three gene signatures were identified, the strongest being a 2-gene combination of *BCL2-CDKN1A*, exhibiting an accuracy of 75% for prediction of outcome. Independent examination using 4 previously-reported microarray datasets of Tamoxifen-treated patient samples (n = 503) confirmed the potential of *BCL2-CDKN1A*. The predictive value was further determined by comparing the ability of the genes to predict recurrence in an additional, previously-published, cohort consisting of Tamoxifen-treated (n = 58, p = 0.015) and untreated patients (n = 62, p = 0.25).

**Conclusions/Significance:**

A novel gene expression signature predictive of outcome of Tamoxifen-treated patients was identified. The validation suggests that *BCL2-CDKN1A* exhibit promising predictive potential.

## Introduction

For patients with breast tumors expressing the estrogen receptor alpha protein (ER+) adjuvant anti-estrogen treatment with Tamoxifen significantly reduce the risk of recurrence and death in all age groups studied. A meta-analysis of 21,457 women with breast cancer included in 20 trials of adjuvant Tamoxifen therapy showed a reduction of 15-year breast cancer mortality rates by at least one third [1]. Women with ER-negative disease exhibited no benefit from the treatment [2]. More recent drug development has lead to third generation aromatase inhibitors (AIs) that have shown increased efficacy compared to Tamoxifen in post-menopausal women [3]–[5]. Tamoxifen remains the treatment modality for pre-menopausal breast cancer patients and patients resistant to AIs. In addition, the various side-effects prevent some patients from receiving AIs [6]–[8]. Furthermore, the majority of patients in many countries receive sequential treatment, e.g. a total of 5 years of endocrine treatment, half on Tamoxifen and half on an AI [9]. Therefore, it is reasonable to continue the study of Tamoxifen as an adjuvant treatment.

The expression of selected genes could provide important markers for predicting outcome in ER+ tumors. A few gene expression signatures have recently emerged that are associated with benefit of Tamoxifen [10]–[14]. Furthermore, the rationale for using gene expression as a clinical tool is emphasized by the fact that two recently developed and validated assays are presently being employed as stratifiers in clinical trials, i.e. TAILORx and MINDACT, which investigates the Oncotype Dx asssay and the 70-gene signature, respectively [15], [16]. A recent review of the five most investigated multi-gene expression based profiles discus the main common ground on which they all refer to, i.e. proliferation-related genes [17].

In this study, we aimed to identify novel gene signatures that accurately predict the outcome of ER+ breast cancer patients who received adjuvant Tamoxifen mono-therapy using quantitative-PCR (qPCR). We employed a matched study design to challenge the difficulty in identifying resistance-specific gene signatures (vs. proliferative), examining the expression of a panel of 59 genes in tumor samples from high-risk, post-menopausal, ER+ patients who had received Tamoxifen as adjuvant mono-therapy. The finding of a 2-gene combination was evaluated in independent patient populations treated with adjuvant Tamoxifen to confirm the ability of the genes to predict outcome. Furthermore, the predictive vs. prognostic potential was examined in an independent cohort of both Tamoxifen-treated and untreated patients.

## Materials and Methods

### Ethics Statement

The study was approved by the Ethical Committee of Funen and Vejle County (VF20040064), The Danish Data Protection Agency (2009-41-3928) and the DBCG. The study was retrospective and we did not obtain informed consent from the participants involved in the study as approved by the Ethical Committee.

### Patient Material

All patients had received adjuvant Tamoxifen as mono-therapy and were extracted from the endocrine protocols, arm C, of the Danish Breast Cancer Co-operative Group (DBCG) 89 and 99 programs [18], being diagnosed no later than Feb. 2002 and having archival frozen tumor tissue (stored at −80°C). Clinical information was obtained from DBCG. Inclusion: ER+ tumor, post-menopausal, treated with Tamoxifen for >3 months, tumor content >50% (haematoxylin- and eosin (HE)-stained cryosections). Exclusion: bilateral breast cancer, recurrence <3 months of diagnosis, treatment with adjuvant chemotherapy/AIs, secondary cancers (except for cancer cutis) or/and unavailable medical records. Recurrence was defined as a clinically-verified metastasis in distant organs. Follow-up was defined as time between diagnosis and date of last flow sheet for patients without recurrence, whereas patients with recurrence were censored at date of recurrence.

### Study Design

Patients (N = 108) were selected nationwide. The criteria for matching patient pairs were based on the Nottingham prognostic index (NPI) [19]. The following 5 criteria were mandatory: 1) lymph node-negative (0) or -positive sub-grouped as follows: 1, 2, 3 or >4 metastatic axillary lymph nodes; 2) tumor size: ≤20 mm or >20 mm; 3) histological diagnosis: ductal or lobular invasive breast carcinomas; 4) malignancy grade: 1, 2 or 3 (only graded for invasive ductal carcinomas); 5) duration of Tamoxifen: if ≤3 years; the treatment period could not differ by >6 months or, if both patients were treated >3 years, all treatment durations were acceptable within the matched pair. In addition, follow-up of the paired patient without recurrence had to be at least equal to the time-to-recurrence of the matched patient with recurrence. Due to the matched study design, the limiting factor for inclusion of patients was the characteristics of the patients with recurrence. Clinical characteristics are listed in Table 1 and the data was collectively analyzed unless otherwise mentioned. We follow the REMARK criteria; however as this is a matched study we cannot fully comply with regards to the data analysis (e.g. uni- and multi-variate analysis).

**Table 1:** Characteristics of patients and their tumor included in the study.

	Non-recurrent	Recurrent
	(n = 54)	(n = 54)
**Age**	**63.3**	**58.7**
Average (range), years	**(49–74)**	**(48–73)**
**Size**	**27**	**30.4**
Average (range), mm	**(12–60)**	**(11–90)**
ER status*		
Positive	53	**52**
Negative	0	**0**
**Unknown**	1	**2**
**PgR status** a		
Negative	35	**35**
Positive	10	**7**
Unknown	9	**2**
**HER2 status** b		
Amplified	2	**3**
Normal	28	**26**
Unknown	24	**25**
**Positive lymph nodes**	**4**	**4.4**
Average (range)	**(0–13)**	**(0–15)**
**Grade**		
1	9	10
2	29	28
3	11	11
Unknown	5	5
**Tamoxifen**	**2.9**	2.6
Average (range), years	**(0.7–7.7)**	(0.3–6.3)
**Time to recurrence**		**4.3**
Avg (range), years	**_**	**(0.7–10.4)**

a Cut-off: ≥1% staining of tumor cells was denoted positive.

b HER2 amplification was investigated by both IHC and FISH.

### Selection of Genes

The 59 candidate genes investigated were selected based on an extensive literature study using the PubMed database [20]. Details are provided in Supplementary material S1 and S2.

### Purification and Evaluation of RNA

Total RNA was purified from a maximum of 35×10 µm cryosections by Roche RNA isolation kits for tissue (MagNa Pure LC RNA isolation kit III tissue, Roche, Basel, Switzerland) using the MagNa Pure Robot (Roche). RNA concentration and purity was examined using the NanoDrop Spectrophotometer (Thermo Scientific, Wilmington, DE, USA). Samples were excluded from further analysis if the concentration was <10 ng/µL and/or if the purity ratio 260/280 was <1.8. The BioAnalyzer 2100 (Agilent Technologies, CA, USA) was used to evaluate samples from different centers. The average RNA integrity number (RIN) was 8.1 (range 6.4–9.5).

### cDNA Synthesis

RNA (10 µL) was reverse-transcribed to cDNA using random 9-mer oligonucleotide primers at 25 µM/reaction. RNA and primers were incubated for 5 min/70°C, placed on ice, and a reaction mixture of 1 mM dNTPs, 1 Unit/µL RNase Inhibitor (Roche), 10 Unit/µL Reverse Transcriptase (Invitrogen Life Technologies, Paisley, UK) and First Strand Buffer x5 (Invitrogen) was added. The material was incubated for 10 min/25°C, followed by 45 min/37°C, and finally 5 min/95°C.

### qPCR/Low Density Arrays

TaqMan® Gene Expression Assays (Applied Biosystems (AB), Foster City, CA, USA) on Low Density Arrays (LDAs) were run for 2 min/50°C, 10 min/94.5°C, followed by 50 cycles of 30 sec/97°C and 1 min/59.7°C. All samples were run in triplicate on the ABI 7900HT system (AB) as technical replicates. The genes listed in Supplementary material S2 were investigated in the first phase using a 63+1 LDA configuration (n = 60). In the second phase (n = 48), an LDA configuration of 31+1 was used to investigate the genes identified in the first phase (n = 18) with p<0.15 (Wilcoxon signed rank sum test). The LDA configuration is pre-defined which left space for 9 additional genes. The resulting 27 genes investigated are marked with an asterisk in Supplementary material S2.

### Data Preparation

qPCR raw data (Supplementary material S3) were analyzed by SDS vers. 2.2 (AB). Criteria for objective removal of outliers: Ct<30: replicates must be within 0.5 Ct of each other, 30≤Ct≤33: replicates must be within 1.0 Ct of each other and 33≤Ct<37: all replicates were included. The Ct value for each target gene was determined by averaging the replicates. Measurements above Ct = 37 were regarded as immeasurable. Target gene Ct-values were normalized to the average of 4 reference genes previously identified (*TBP, RPLP0, PUM1* and *ACTB*) [21], thereby obtaining the ΔCt (ΔCt_target_ = Ct_target_ - Ct_ref,avg_). The difference in gene expression for a given target gene between the matched patient-pairs: ΔΔCt = ΔCt_recurrent_ - ΔCt_non-recurrent_. In case one of the patients’ ΔCt values were immeasurable, the ΔΔCt was computed using a value of 40 for the missing value. If the ΔCt value was immeasurable for both patients in a pair, no ΔΔCt value was computed. The primary endpoint for all statistical analyses was time from primary surgery to recurrence.

### Statistics – qPCR Data

The Wilcoxon singed rank sum test was used to assess the differential gene expression between patients and the overall significance considered Bonferroni corrected p-values.

Conditional logistic regression was used to determine optimal pairs, triples and quadruples of genes. For the optimal model, we determined the rate of correct classification, i.e. accuracy, using cross validation, leave-one-pair-out. Additionally, a non-parametric bootstrap, based on 1000 x re-sampling of the pairs, was used to determine the stability. The 27 genes analyzed for all 54 patient-pairs were used in model building, cross validation and the bootstrap method. In addition, the genes were subjected to modified microarray-based statistics (Statistical Analysis of Microarray (SAM)) [22].

The *HOXB13:IL17BR* ratio was investigated analogous to previously reported [11]. Twelve pairs with undetermined values for *HOXB13* in both patients were excluded, as this would lead to an estimate of the effect of *IL17BR* alone.

All statistical computations were conducted in Stata vs.10.1 (StataCorp, TX, USA) unless otherwise mentioned.

### Statistics - Microarray Datasets

Four previously-published microarray datasets [11]–[14], investigating patient samples treated with Tamoxifen and with characteristics similar to ours were used for independent examination. Complete lists of our three signatures relative to the microarray datasets are provided in the Supplementary material S4. One of the studies included data from 3 microarray platforms [12] and another included data from 2 [11], giving a total of 7 platforms for validation. One of these platforms (GSE6532-GPL97) was missing 15/27 genes, and was excluded from further analysis. The identified genes from the qPCR analysis were annotated to the probe IDs using Gene Symbol (Supplementary material S4). The average detection of probes was used. Each signature-gene selected by the modified SAM procedure [22] was submitted to SVM. Performance of the selected gene signatures was assessed by leave-one-pair-out validation to obtain the accuracy, sensitivity and specificity. In all analyses of recurrence data, the mean accuracy was calculated as (number of patients with recurrence predicted as recurrent patients+number of patients without recurrence predicted as non-recurrent patients)/total number of patients). The cut-off threshold distinguishing recurrent from non-recurrent patients was calculated as the proportion of tumors from patients with recurrence in the total sample. Furthermore, univariate and multivariate analyses were performed using Cox regression models. The Cox regression models estimate relative risks (RR) with confidence intervals (CI) for the prediction signature and clinical variables. The Kaplan-Meier plot was used to show differential survival in the predicted groups (Supplementary material S5).

Upon examination of the performance of the signatures in the four previously-published datasets mentioned above, *BCL2-CDKN1A* was determined to be the most promising. This signature was then evaluated for prognostic vs. predictive capabilities using survival statistics in a different dataset (GSE2990 [23]). Patient samples from the dataset were included in the analysis if they were >50 years and had an ER+ tumor, resulting in the following tumor characteristics: untreated patients were all N- and 41% of the tumors were ≥20 mm, while the Tamoxifen-treated patients were 57% N+ and 61% had tumors ≥20 mm. Kaplan-Meier curves are used to show their differential survival with a p-value from Chi-square test, constructed after adjusting for clinical variables. In addition, both univariate and multivariate Cox regression analyses were conducted to investigate the RR for the 2-gene prediction signature.

## Results

### Patient Material

Tumors from patients with high-risk, post-menopausal, ER+ breast cancer treated only with adjuvant Tamoxifen [18] were investigated, all diagnosed before 2002. A nested matched case-control study design was used to increase the likelihood of identifying genes associated with outcome beyond the parameters used for matching. The median clinical follow-up was 4.8 years (range 0.7–10.4 years; censored at time of recurrence or at the last date of clinically verified recurrence-free). The patient and tumor characteristics are provided in Table 1.

### Analysis of Single Genes

The gene expression of a panel of 59 genes was examined by qPCR (raw data provided in Supplementary material S3). The 59 genes were selected based on previously published studies where they were associated with outcome after Tamoxifen treatment, while genes uniformly associated with prognosis were excluded (Supplementary material S1 and S2). Gene expression values were obtained for 98% of all genes, across all samples. The remaining 2% could reasonably be assumed to be genes not expressed since the reference genes were adequately expressed. Comparative analysis of the genes according to altered expression across the recurrent vs. non-recurrent patient samples (Table 2) identified *BCL2* as the most significant (p = 0.0002), and it remained significant upon Bonferroni correction.

**Table 2:** The most significant genes exhibiting altered expression in the recurrent vs. non-recurrent patient samples identified using single gene analysis, p<0.1.

Ranking	gene	p_wil
1	*BCL2*	0.0002
2	*PRKCE*	0.0104
3	*PRKCD*	0.015
4	*NRG1*	0.0185
5	*EGFR*	0.0228
6	*NCOA1*	0.0384
7	*ESR1*	0.0409
8	*IGF1R*	0.0453
9	*CDKN1A*	0.0699
10	*TNF*	0.0812
11	*RARA*	0.089
12	*XBP1*	0.0907

The genes are ranked by the p-value of the Wilcoxon signed rank test (p_wil).

### Construction of Gene Combinations

**Figure 1:**
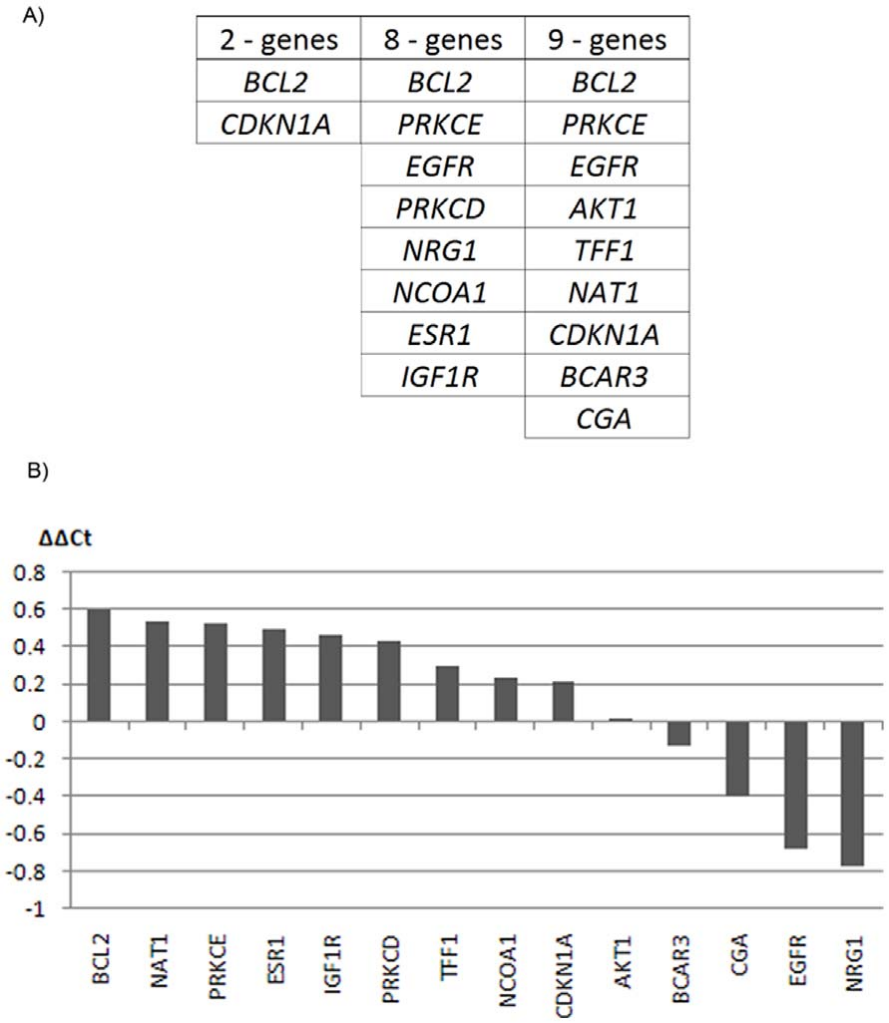
Genes identified and their expression pattern. A) The 2-, 8- and 9-gene signatures identified by various statistical analyses. B) ΔΔCt of the genes present in the 2-, 8- and 9-gene signatures. *BCL2* overlap in all three, whereas *CDKN1A* is in the 2- and 9-gene signatures, and *PRKCE* and *EGFR* are in both the 8- and 9-gene signatures. A positive ΔΔCt_median_ value denote that the expression of the gene is highest in the tumor sample from patients without recurrence, whereas a negative value means the expression is higher in the tumor samples from patients with recurrence.

**Figure 2:**
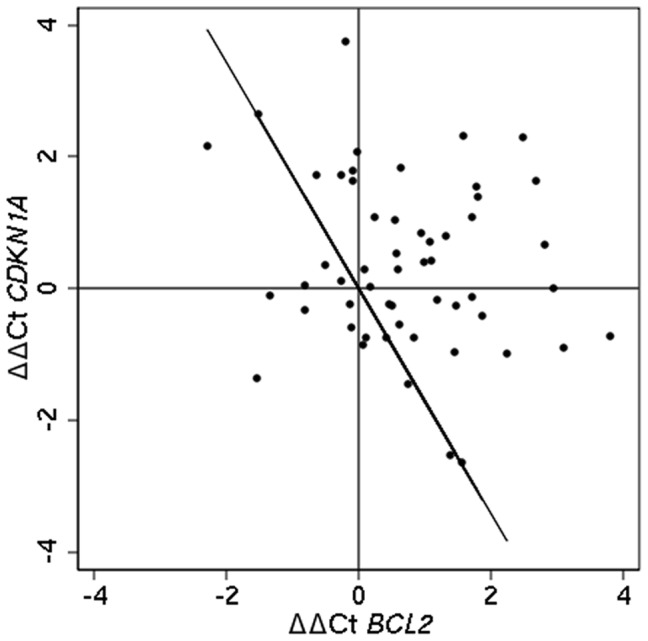
Joint distribution of the ΔΔCt values of *BCL2* and *CDKN1A*. The diagonal line corresponds to the rule determined by conditional logistic regression. Pairs to the right of the line are correctly classified with respect to their outcome (recurrence/non-recurrence) (accuracy of 75%), whereas pairs left of the line are classified incorrectly.

Cross validation was used to determine accuracy and the optimal combination for predicting outcome was identified to be the two-genes *BCL2-CDKN1A* (accuracy = 75%), whereas the optimal 3-gene combination consisted of *BCL2-CDKN1A-NAT1* (accuracy = 55%) (Fig. 1A). *BCL2* alone had an accuracy of 70%. The accuracy of the best-performing combination of *BCL2-CDKN1A* is depicted in Fig. 2. The statistical stability of *BCL2-CDKN1A* was found to be sub-optimal as the 2 genes were found to rank highest in only 24.3% of the 1000 bootstrap sampling.

A modified microarray-based statistical analysis based on a machine-learning procedure combining a modified SAM analysis and SVM, identified a 9-gene signature. This 9-gene signature enabled correct classification of 73% of the patient-pairs with regards to recurrence. A list of the identified genes is provided in Fig. 1A along with the direction of expression (Fig. 1B), which was observed to primarily be higher in the tumors of patients that had not developed recurrence.

### Analysis of the *HOXB13: IL17BR* Ratio as Predictive Score

The *HOXB13:IL17BR* ratio has been reported to predict outcome in early breast cancer patients treated with Tamoxifen [11], thus we evaluated the predictive value of this ratio in our data set using the same approach as previously reported. As found by Ma et al. (2004) [11], *HOXB13* showed higher expression in tumor samples from patients with recurrence, and *IL17BR* had higher expression in tumor samples from patients without recurrence. The *HOXB13:IL17BR* ratio correctly classified 64%, and the Wilcoxon signed rank sum test, applied to the ratio values, yielded a p-value of 0.02. The study by Ma et al (2004) was mainly based on early stage cancers with few tumor-infiltrated lymph nodes, whereas our patient population consisted mainly of patients with several tumor-infiltrated lymph nodes at time of diagnosis (average was 4, and only 3/54 pairs had no tumor-infiltrated lymph nodes). Indeed, the predictive value of the ratio was higher in the 21 pairs with a maximum of 3 affected lymph nodes (71% correctly classified, p = 0.03) compared to the 21 pairs with >3 affected lymph nodes (57%, p = 0.29).

### Validation in Independent Microarray Datasets of Tamoxifen-treated Patient Samples

The identified 2-, 8-, and 9-gene expression signatures were examined for predictive capabilities in 6 microarray datasets (Supplementary material S4) from four previously published studies (Table 3) [11]–[14].

**Table 3:** Summary of the four previously-published gene expression datasets examining samples from patients treated with adjuvant Tamoxifen and used for examination of our 3 gene signatures.

No. of patients	GSE accession number	Reference
n = 60	GSE1378	Ma et al. (2004) [11]
	GSE1379	
n = 152^a^	GSE6532	Loi et al. (2008) [12]
n = 155	GSE 9893	Chanrion et al. (2008) [13]
n = 136	GSE12093	Zhang et al. (2009) [14]

a A total of 255 patient samples were analyzed by Loi et al. [12], but only 152 samples were included in our analysis. The remaining 103 patients were either not coupled to clinical data, were not treated with Tamoxifen or analyzed using platform GPL97 (one of three platforms used in this study), which was excluded since 79% of the probes for the genes of interest were missing.

**Figure 3:**
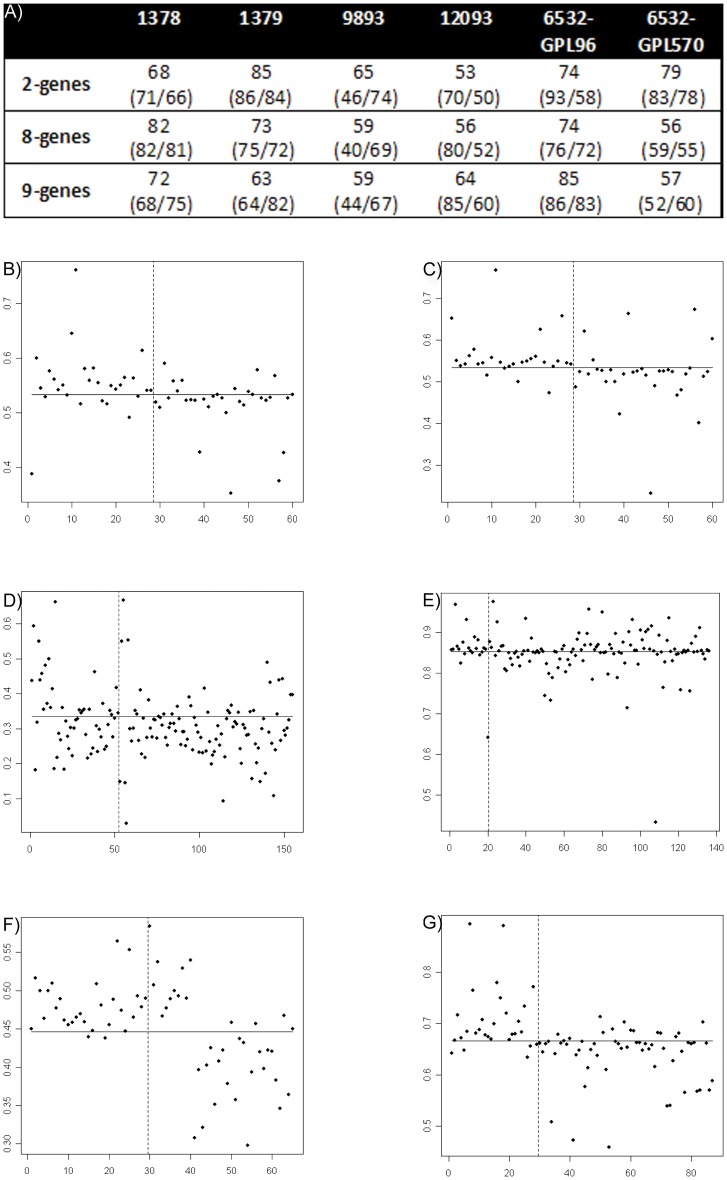
Performance of the identified genes. The capabilities of the identified 2-, 8- and 9-gene signatures to predict recurrence was evaluated in 6 independent gene expression datasets. A) Summarized results of accuracy (%), along with sensitivity/specificity in parenthesis (both given as %), of the identified signatures to predict recurrence. B–G) Dot-plots of the identified 2-gene signature (*BCL2-CDKN1A)* illustrating the probability of recurrence. The vertical line separates the cases, i.e. patients with recurrence (left of the line) from controls (right of the line). The horizontal line refers to the cut-point used, hence the upper left and lower right corners includes the correctly classified patients. X-axis denotes the patient index in the study (same random order as original study). The Y-axis is the SVM probability of recurrence. B) GSE1378 C) GSE1379 D) GSE9893 E) GSE12093 F) GSE6532-GPL96 and G) GSE6532-GPL570.

Overall, the accuracies for the 2-, 8- and 9-gene signatures were high (Fig. 3A), especially for the 2-gene signature, *BCL2-CDKN1A*, with half of the platforms having an accuracy of >70% (Fig. 3B, C, D, E, F, G). One of the studies also investigated tumor tissue from matched patient material (GSE1379), similar to our study design. The 2-gene signature performed even better in this independent population, exhibiting an accuracy of 85%vs. 75% in our qPCR dataset. Furthermore, in this microarray dataset (GSE1379), which led to the identification of the *HOXB13:IL17BR* ratio [11], *BCL2-CDKN1A* challenged the *HOXB13:IL17BR* ratio, as *BCL2-CDKN1A* exhibited an accuracy of 85% vs. the reported 81% accuracy for the *HOXB13:IL17BR* ratio.

For the 2- and 8-gene signatures, the sensitivity was higher than the specificity across all studies except GSE9893. This was most pronounced for *BCL2-CDKN1A* in GSE6532-GPL96, which showed the highest sensitivity across all studies (93%). GSE9893 appeared to be quite different from the others in that it had low sensitivities, but fair specificities, for all signatures.

This above-mentioned independent evaluation pointed to the 2-gene signature as the most promising (Fig. 3A), and we therefore conducted univariate and multivariate Cox analyses of these external datasets, including Kaplan-Meier plots. GSE1379 and GSE6532-GPL570 were both significant in the univariate analysis (p<0.01), and the latter remained significant in the multivariate analysis (p = <0.0001), with a relative risk (RR) of 18.30. The findings are collectively shown in Supplementary material S5.

### Prognostic vs. Predictive Value of the *BCL2-CDKN1A* Gene Signature

**Figure 4:**
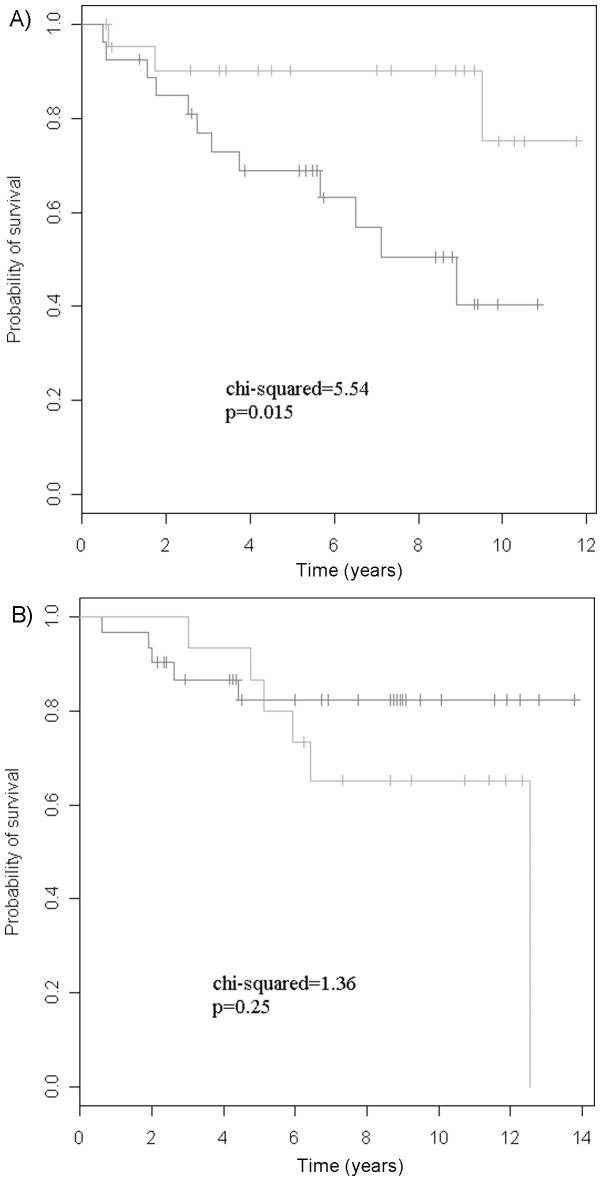
Survival analysis of the 2-gene signature. Kaplan-Meier curves of recurrence-free survival according to model-based prediction of outcome using the 2-gene signature (*BCL2-CDKN1A*) for the independent gene expression dataset GSE2990. Grey line (top) indicates the good outcome signature, whereas the black line (bottom) indicates the poor outcome signature. Only data from post-menopausal (>50 years) and ER+ breast cancer patients were included in the analysis. Data was adjusted for clinical variables. A) Tamoxifen-treated patient samples (N = 58). B) Untreated patient samples (N = 62).

We furthermore conducted survival analysis of the 2 genes in a new, independent, previously-published microarray cohort that contained both treatment-naïve (N = 62) and Tamoxifen-treated (N = 58) patient samples (GSE2990 [23]). Patients included in the analysis were all >50 years of age at diagnosis (i.e. defined as post-menopausal) and ER+. Kaplan-Meier curves were obtained after adjusting for clinical variables. Figure 4 shows that the 2-gene signature could separate the Tamoxifen-treated patients with respect to probability of recurrence-free survival (p = 0.015), while it did not significantly separate the outcome of the untreated patient samples (p = 0.25). This finding was supported by the 2-gene signature being a significant predictor of outcome in both univariate and multivariate Cox regression analysis (both p<0.0001), with a RR of 9.09 (CI: 3.1–26.7) and 64.5 (CI: 10.6–390.7), respectively, in the Tamoxifen-treated patients. The 2-gene signature did not reach significance in either univariate or multivariate analysis of the untreated patients.

## Discussion

Despite Tamoxifen being an effective drug for many ER+ breast cancer patients in the adjuvant setting, about a third will experience recurrence. To address this issue, we investigated the expression of 59 genes in early, high-risk, ER+, post-menopausal, breast cancer patients receiving adjuvant Tamoxifen mono-therapy.

Single-gene analysis revealed 8 genes (p<0.05) that, overall, were found to have higher expression in patients without recurrence than those with. Conditional logistic regression identified an optimal gene–pair, *CDKN1A* and *BCL2*, exhibiting an accuracy of 75%. This 2-gene combination was evaluated in independent patient populations treated with adjuvant Tamoxifen (n = 503), which confirmed the ability of the genes to predict outcome. Furthermore, the predictive (vs. prognostic) potential was confirmed by examination of an independent cohort comprising both Tamoxifen-treated (n = 58, p = 0.015) and untreated patients (n = 62, p = 0.25).

Breast cancer is a versatile disease and several molecular profiles and subgroups have been identified that correlate with outcome (recently reviewed by Eroles et at. [24]). The most comprehensive with regard to biology [24] is the intrinsic subtype model set forth by Perou et al. [25], [26], which identified 6 molecular subgroups, half of which contain ER+ tumors, i.e. the normal breast-like, and the luminal A and B groups. Although the the tumors in our study were not extensively profiled, they predominantly belong to the luminal A subgroup, as they express the PgR and do not show amplification of HER2.

The strategy of selection of the 59 genes included in our study (Supplementary material S2) was very similar to the approach used in the initial study design, which lead to Oncotype Dx assay [10]. Our method diverged in that we chose to exclude genes uniformly identified to be associated with prognosis and solely with the ER+ phenotype to focus on genes that may prove to be purely predictive. This led to the exclusion of several genes associated with the luminal subtypes (as they distinguish between ER+ and ER- and/or HER2 amplified tumors) and *ki-67* (a proliferation-associated gene). Because of this approach, our genes cannot be directly compared with known profiles such as the Oncotype Dx [10] or PAM50 [27], both qPCR assays applied to Tamoxifen-treated populations. As recently discussed by the primary investigator behind Oncotype Dx, these assays primarily provide information on proliferative activity [17]. Though it is not likely that exclusively predictive genes will be identified, our goal was, to eliminate strong prognostic genes that might mask the more subtle predictive genes.

Concurrent with our work, two interesting studies identified several chromosomal loci associated with resistance towards Tamoxifen, harboring many of the genes investigated in this study, which supports our approach. The first study identified, among others, the loci of 17q12 and 17q21.33-q25.1 [28], whereas a hypothesis-generating study from 2012 of 2,000 breast cancer samples identified the loci of 17q23/q20 (in an intermediate prognosis group of predominantly ER+ tumors) and 11q13/14 (in an ER+-subgroup with elevated mortality hazard ratios) [29].

To further increase the likelihood of identifying genes with predictive capabilities, we employed a nested matched case-control study design [11], [30]–[32]. Matching of patients was carefully conducted to avoid confounding or overmatching, a design that highlights the association being examined (i.e. recurrence despite treatment) [31], [33], [34]. The patients were selected and matched solely according to clinical characteristics, as these parameters are the only internationally recognized and applied stratifiers guiding clinicians in the outcome of ER+ breast cancer patients.

Other studies have also found a correlation between low expression of *BCL2* and lack of response to Tamoxifen [10], [35]–[38], as found in our study. This association of *BCL2* and favorable outcome may relate to the fact that *BCL2* is an estrogen-regulated gene [39], thus indicative of an intact pathway driving tumor growth and thereby sensitivity to Tamoxifen. In addition, *BCL2* is part of the Oncotype Dx assay [40]. The full potential and underlying biology of *BCL2* as a prognostic and/or predictive value remains to be determined, although it is clearly a promising marker [41].

The other gene in the 2-gene signature was *CDKN1A*, cyclin-dependent kinase inhibitor 1A, which encodes the protein p21^WAF1/CIP1^; the increased expression of which also previously has been found to be specifically associated with good outcome after treatment with Tamoxifen [42]. p21^WAF1/CIP1^ has also been reported to be absent in a clinical case of Tamoxifen-stimulated growth [43]. p21^WAF1/CIP1^ interacts with several cell cycle regulators, but the precise mechanism(s) behind its role in Tamoxifen resistance remains to be elucidated.

The gene combination of *BCL2-CDKN1A* outperformed the 8- and 9-gene signatures, showing an increased ability to correctly classify patients with recurrence despite Tamoxifen treatment, with an accuracy of 75%. The microarray studies investigated patients with varying numbers of tumor-infiltrated lymph nodes at the time of diagnosis, which is known to be a strong parameter associated with outcome. The GSE12093 study, which examined only patients with lymph node-negative tumors, and GSE9893 with few N+-patients, had the poorest accuracies, as expected, since the tumors we used for signature identification were from patients with many tumor-infiltrated lymph nodes (average of 4). On the other hand, the study with many N+-patients resulted in accuracies of 74% and 79%, respectively, which was very similar to ours (GSE6532; GPL96 and GPL570). A higher accuracy (85%) was observed for the dataset investigating matched patient material according to TNM-stage and grade, which was nearly identical to our study design (GSE1379).These findings underscore the fact that clinical variables, especially nodal involvement, even within the same cancer sub-types, are important, and it confirms the theory that different biomarkers are needed for high- vs. low-risk patients.

The importance of the clinical parameters of nodal status was further supported by investigating the 2-gene ratio of *HOXB13:IL17BR* [11], [44], which was developed from tumors from patients with few affected lymph nodes, in our dataset. We investigated our complete dataset and found that the ratio correctly classified 64% of the patients, but separating our dataset into patients having either ≤3 or >3 lymph nodes, we found the ratio could correctly classify 71% and 57%, respectively.

Since only patients treated with Tamoxifen were used to develop the signature in this study, it was not possible to unequivocally distinguish whether the identified genes encompassed prognostic or/and predictive properties. However, matching with known prognostic factors implied that the genes identified as being associated with outcome provide information beyond the factors used for matching. Moreover, our demonstration that the most promising signature consisting of *BCL2-CDKN1A* could not significantly separate untreated patient samples according to outcome, but significantly separated Tamoxifen-treated patient samples in an independent dataset, supports it being a predictive signature. It should be noted that the patients in the untreated dataset were all N- since ethical considerations preclude denying treatment to N+ patients (the compared treated dataset was 57% N+). Otherwise, the dataset is directly comparable to our study. Furthermore, as this is a hypothesis-generating study employing previously published microaarray datasets for verification, the 2-gene signature will be experimentally validated in a large independent cohort using qPCR to determine the clinical value and compare the 2-gene signature with existing profiles. Furthermore, the underlying biology of the 2 genes, and their association with various pathways in the ER+ cell, should be investigated.

Gene profiles will very likely expand in use, aiding clinical treatment decisions and leading to increasingly individualized treatment strategies [17], [45], [46] that include clinical characteristics, such as tumor size, grade and lymph node involvement, with the proteins expressed by the tumors (immunohistochemical (IHC) analysis), such as the ER, ki-67, HER2, and molecular profiles. A combinational strategy using both clinical characteristics and protein expression in an algorithm has been set forth by the Adjuvant! Online program [47]–[49] and the IHC4+ profile [50], incorporating parameters from the nearly 30 year old NPI [19], [51] and recognized IHC markers (ER, PgR, ki-67 and HER2). These algorithms still need some adjustments and validation, but future clinical algorithms will likely include molecular profiles.

In summary, we identified a 2-gene signature, *BCL2-CDKN1A*, which was, upon evaluation in independent datasets, found to be a potential predictor of outcome for high-risk ER+ breast cancer patients treated with Tamoxifen.

## Supporting Information

Supplementary Material S1 References for the selection of the 59 candidate genes investigated.
(DOCX)

Supplementary Material S2 Details of the candidate genes selected for investigation.
(DOC)

Supplementary Material S3 Table of the raw Ct values.
(XLSX)

Supplementary Material S4 Identified Genes and corresponding probes in previously-published microarray datasets.
(DOC)

Supplementary Material S5 Cox regression analysis of the independent datasets.
(DOCX)
