# Supplementary material for: Gene Expression Signatures That Predict Outcome of Tamoxifen-Treated Estrogen Receptor-Positive, High-Risk, Primary Breast Cancer Patients: A DBCG Study
Source: PLoS One. 2013 Jan 16;8(1):e54078. doi: 10.1371/journal.pone.0054078 (PMC3546921; doi:10.1371/journal.pone.0054078)
Supplement: Supplementary Material S1 — References for the selection of the 59 candidate genes investigated. (DOCX) [file pone.0054078.s001.docx]

**Supplementary material S1**

*Selection of genes*

The 59 candidate genes investigated were selected based on an extensive literature study (Jan. 2006) using the PubMed literature database [1] with the search criteria: “breast cancer Tamoxifen”, and either “resistance” (yielding 779 papers) or “prediction” (yielding 82 papers). Only papers reporting gene expression analysis of patient tumor samples were included (n=79) [2–43]. Genes uniformly reported to be associated with prognosis, such as the proliferation marker *Ki-67,* were excluded; whereas any gene reported to be associated with resistance/prediction of outcome in Tamoxifen-treated patients were included. The 40 remaining papers served as the basis of the selected genes.

**References**

1. NCBI (2005) PubMed. webpage: pubmed gov .

2. Arpino G, Weiss H, Lee AV, Schiff R, De PS, et al. (2005) Estrogen receptor-positive, progesterone receptor-negative breast cancer: association with growth factor receptor expression and tamoxifen resistance. J Natl Cancer Inst 97: 1254-1261.

3. Berstein LM, Zheng H, Yue W, Wang JP, Lykkesfeldt AE, et al. (2003) New approaches to the understanding of tamoxifen action and resistance. Endocr Relat Cancer 10: 267-277.

4. Bieche I, Parfait B, Nogues C, Andrieu C, Vidaud D, et al. (2001) The CGA gene as new predictor of the response to endocrine therapy in ER alpha-positive postmenopausal breast cancer patients. Oncogene 20: 6955-6959.

5. Bieche I, Girault I, Urbain E, Tozlu S, Lidereau R (2004) Relationship between intratumoral expression of genes coding for xenobiotic-metabolizing enzymes and benefit from adjuvant tamoxifen in estrogen receptor alpha-positive postmenopausal breast carcinoma. Breast Cancer Res 6: R252-R263.

6. Bouras T, Southey MC, Venter DJ (2001) Overexpression of the steroid receptor coactivator AIB1 in breast cancer correlates with the absence of estrogen and progesterone receptors and positivity for p53 and HER2/neu. Cancer Res 61: 903-907.

7. Britton DJ, Hutcheson IR, Knowlden JM, Barrow D, Giles M, et al. (2006) Bidirectional cross talk between ERalpha and EGFR signalling pathways regulates tamoxifen-resistant growth. Breast Cancer Res Treat 96: 131-146.

8. Brockdorff BL, Heiberg I, Lykkesfeldt AE (2003) Resistance to different antiestrogens is caused by different multi-factorial changes and is associated with reduced expression of IGF receptor Ialpha. Endocr Relat Cancer 10: 579-590.

9. Butt AJ, McNeil CM, Musgrove EA, Sutherland RL (2005) Downstream targets of growth factor and oestrogen signalling and endocrine resistance: the potential roles of c-Myc, cyclin D1 and cyclin E. Endocr Relat Cancer 12 Suppl 1: S47-S59.

10. Ciocca DR, Elledge R (2000) Molecular markers for predicting response to tamoxifen in breast cancer patients. Endocrine 13: 1-10.

11. Clarke R, Skaar TC, Bouker KB, Davis N, Lee YR, et al. (2001) Molecular and pharmacological aspects of antiestrogen resistance. J Steroid Biochem Mol Biol 76: 71-84.

12. Clarke R, Liu MC, Bouker KB, Gu Z, Lee RY, et al. (2003) Antiestrogen resistance in breast cancer and the role of estrogen receptor signaling. Oncogene 22: 7316-7339.

13. Daidone MG, Luisi A, Martelli G, Benini E, Veneroni S, et al. (2000) Biomarkers and outcome after tamoxifen treatment in node-positive breast cancers from elderly women. Br J Cancer 82: 270-277.

14. de Cremoux P, Tran-Perennou C, Brockdorff BL, Boudou E, Brunner N, et al. (2003) Validation of real-time RT-PCR for analysis of human breast cancer cell lines resistant or sensitive to treatment with antiestrogens. Endocr Relat Cancer 10: 409-418.

15. Gee JM, Willsher PC, Kenny FS, Robertson JF, Pinder SE, et al. (1999) Endocrine response and resistance in breast cancer: a role for the transcription factor Fos. Int J Cancer 84: 54-61.

16. Girault I, Lerebours F, Amarir S, Tozlu S, Tubiana-Hulin M, et al. (2003) Expression analysis of estrogen receptor alpha coregulators in breast carcinoma: evidence that NCOR1 expression is predictive of the response to tamoxifen. Clin Cancer Res 9: 1259-1266.

17. Gu Z, Lee RY, Skaar TC, Bouker KB, Welch JN, et al. (2002) Association of interferon regulatory factor-1, nucleophosmin, nuclear factor-kappaB, and cyclic AMP response element binding with acquired resistance to Faslodex (ICI 182,780). Cancer Res 62: 3428-3437.

18. Jansen MP, Foekens JA, van S, I, rkzwager-Kiel MM, Ritstier K, et al. (2005) Molecular classification of tamoxifen-resistant breast carcinomas by gene expression profiling. J Clin Oncol 23: 732-740.

19. Keeton EK, Brown M (2005) Cell cycle progression stimulated by tamoxifen-bound estrogen receptor-alpha and promoter-specific effects in breast cancer cells deficient in N-CoR and SMRT. Mol Endocrinol 19: 1543-1554.

20. Kenny FS, Hui R, Musgrove EA, Gee JM, Blamey RW, et al. (1999) Overexpression of cyclin D1 messenger RNA predicts for poor prognosis in estrogen receptor-positive breast cancer. Clin Cancer Res 5: 2069-2076.

21. Knoop AS, Bentzen SM, Nielsen MM, Rasmussen BB, Rose C (2001) Value of epidermal growth factor receptor, HER2, p53, and steroid receptors in predicting the efficacy of tamoxifen in high-risk postmenopausal breast cancer patients. J Clin Oncol 19: 3376-3384.

22. Knowlden JM, Hutcheson IR, Jones HE, Madden T, Gee JM, et al. (2003) Elevated levels of epidermal growth factor receptor/c-erbB2 heterodimers mediate an autocrine growth regulatory pathway in tamoxifen-resistant MCF-7 cells. Endocrinology 144: 1032-1044.

23. Knowlden JM, Hutcheson IR, Barrow D, Gee JM, Nicholson RI (2005) Insulin-like growth factor-I receptor signaling in tamoxifen-resistant breast cancer: a supporting role to the epidermal growth factor receptor. Endocrinology 146: 4609-4618.

24. List HJ, Reiter R, Singh B, Wellstein A, Riegel AT (2001) Expression of the nuclear coactivator AIB1 in normal and malignant breast tissue. Breast Cancer Res Treat 68: 21-28.

25. Ma XJ, Wang Z, Ryan PD, Isakoff SJ, Barmettler A, et al. (2004) A two-gene expression ratio predicts clinical outcome in breast cancer patients treated with tamoxifen. Cancer Cell 5: 607-616.

26. Meijer-van Gelder ME, Look MP, Peters HA, Schmitt M, Brunner N, et al. (2004) Urokinase-type plasminogen activator system in breast cancer: association with tamoxifen therapy in recurrent disease. Cancer Res 64: 4563-4568.

27. Miller WR, Hulme MJ, Bartlett JM, MacCallum J, Dixon JM (1997) Changes in messenger RNA expression of protein kinase A regulatory subunit ialpha in breast cancer patients treated with tamoxifen. Clin Cancer Res 3: 2399-2404.

28. Murphy LC, Leygue E, Niu Y, Snell L, Ho SM, et al. (2002) Relationship of coregulator and oestrogen receptor isoform expression to de novo tamoxifen resistance in human breast cancer. Br J Cancer 87: 1411-1416.

29. Nabha SM, Glaros S, Hong M, Lykkesfeldt AE, Schiff R, et al. (2005) Upregulation of PKC-delta contributes to antiestrogen resistance in mammary tumor cells. Oncogene 24: 3166-3176.

30. Nicholson RI, Hutcheson IR, Knowlden JM, Jones HE, Harper ME, et al. (2004) Nonendocrine pathways and endocrine resistance: observations with antiestrogens and signal transduction inhibitors in combination. Clin Cancer Res 10: 346S-354S.

31. Osborne CK, Bardou V, Hopp TA, Chamness GC, Hilsenbeck SG, et al. (2003) Role of the estrogen receptor coactivator AIB1 (SRC-3) and HER-2/neu in tamoxifen resistance in breast cancer. J Natl Cancer Inst 95: 353-361.

32. Osborne CK, Schiff R (2005) Estrogen-receptor biology: continuing progress and therapeutic implications. J Clin Oncol 23: 1616-1622.

33. Paik S, Shak S, Tang G, Kim C, Baker J, et al. (2004) A multigene assay to predict recurrence of tamoxifen-treated, node-negative breast cancer. N Engl J Med 351: 2817-2826.

34. Pompeo F, Brooke E, Kawamura A, Mushtaq A, Sim E (2002) The pharmacogenetics of NAT: structural aspects. Pharmacogenomics 3: 19-30.

35. Ring A, Dowsett M (2004) Mechanisms of tamoxifen resistance. Endocr Relat Cancer 11: 643-658.

36. Saji S, Hirose M, Toi M (2005) Clinical significance of estrogen receptor beta in breast cancer. Cancer Chemother Pharmacol 56 Suppl 1: 21-26.

37. Schiff R, Massarweh S, Shou J, Osborne CK (2003) Breast cancer endocrine resistance: how growth factor signaling and estrogen receptor coregulators modulate response. Clin Cancer Res 9: 447S-454S.

38. Speirs V, Malone C, Walton DS, Kerin MJ, Atkin SL (1999) Increased expression of estrogen receptor beta mRNA in tamoxifen-resistant breast cancer patients. Cancer Res 59: 5421-5424.

39. Stendahl M, Kronblad A, Ryden L, Emdin S, Bengtsson NO, et al. (2004) Cyclin D1 overexpression is a negative predictive factor for tamoxifen response in postmenopausal breast cancer patients. Br J Cancer 90: 1942-1948.

40. Tovey S, Dunne B, Witton CJ, Forsyth A, Cooke TG, et al. (2005) Can molecular markers predict when to implement treatment with aromatase inhibitors in invasive breast cancer? Clin Cancer Res 11: 4835-4842.

41. Zhong M, Lu Z, Abbas T, Hornia A, Chatakondu K, et al. (2001) Novel tumor-promoting property of tamoxifen. Cell Growth Differ 12: 187-192.

42. Baum M, Budzar AU, Cuzick J, Forbes J, Houghton JH, et al. (2002) Anastrozole alone or in combination with tamoxifen versus tamoxifen alone for adjuvant treatment of postmenopausal women with early breast cancer: first results of the ATAC randomised trial. Lancet 359: 2131-2139.

43. Frogne T, Laenkholm AV, Lyng MB, Henriksen KL, Lykkesfeldt AE (2009) Determination of HER2 phosphorylation at tyrosine 1221/1222 improves prediction of poor survival for breast cancer patients with hormone receptor-positive tumors. Breast Cancer Res 11: R11.
