# Supplementary material for: Gene Expression Signatures That Predict Outcome of Tamoxifen-Treated Estrogen Receptor-Positive, High-Risk, Primary Breast Cancer Patients: A DBCG Study
Source: PLoS One. 2013 Jan 16;8(1):e54078. doi: 10.1371/journal.pone.0054078 (PMC3546921; doi:10.1371/journal.pone.0054078)
Supplement: Supplementary Material S2 — Details of the candidate genes selected for investigation. (DOC) [file pone.0054078.s002.doc]

Supplementary material S2: Overview of the genes investigated in the first phase, n=59, and in the second phase, n=27 (marked with an asterisk (*)). The assay ID denotes manufacturers’ (Applied Biosystems) internal reference ID. Furthermore, the cytoband location is provided. ^¥^: reference genes (n=4).

|  | Primary gene symbol as annotated by manufacturer | Assay ID | Location |
| --- | --- | --- | --- |
|  | CYP19A1 (NM_031226.1,NM_000103.2) | Hs00240671_m1 | 15q21.1 |
| ***** | PRKCD (NM_212539.1,NM_006254.3) | Hs00178914_m1 | 3p21.31 |
|  | NPM3 (NM_006993.1) | Hs00199625_m1 | 10q24.31 |
|  | NCOR2 (NM_006312.2) | Hs00196955_m1 | 12q24 |
|  | EGR1 (NM_001964.2) | Hs00152928_m1 | 5q31.1 |
|  | TGFA (NM_003236.1) | Hs00608187_m1 | 2p13 |
|  | YWHAZ (NM_003406.2) | Hs00237047_m1 | 8q23.1 |
| ***** | PAI1 (NM_000602.1) | Hs00167155_m1 | 7q21.3.q22 |
|  | MYC (NM_002467.3) | Hs00153408_m1 | 8q24.12-.13 |
|  | IGF1 (NM_000618.2) | Hs00153126_m1 | 12q22-q23 |
|  | CDKN1B (NM_004064.2) | Hs00153277_m1 | 12p13.1-p12 |
| **¥** | PUM1 (NM_001020658.1,NM_014676.2) | Hs00206469_m1 | 1p35.2 |
|  | FOS (NM_005252.2) | Hs00170630_m1 | 14q24.3 |
|  | NCOA3 (NM_181659.1,NM_006534.2) | Hs00180722_m1 | 20q12 |
|  | CGA (NM_000735.2) | Hs00174938_m1 | 6q12-q21 |
| ***** | NRG1 (NM_013956.1, NM_013957.1, NM_013958.1, NM_013959.1, NM_013961.1, NM_013962.1) | Hs00247624_m1 | 8p21-p12 |
|  | NCOR1 (NM_006311.2) | Hs00196920_m1 | 17p11.2 |
|  | CCND1 (NM_053056.1) | Hs00277039_m1 | 11q13 |
| ***** | EGFR (NM_005228.3) | Hs00193306_m1 | 7p12 |
| **¥** | TBP (NM_003194.3) | Hs00427620_m1 | 6q27 |
| ***** | IGF1R (NM_000875.2) | Hs00181385_m1 | 15q26.3 |
| **¥** | ACTB (NM_001101.2) | Hs99999903_m1 | 7p15-p12 |
|  | IL6 (NM_000600.1) | Hs00174131_m1 | 7p21 |
|  | NRG2 (NM_004883.1, NM_013981.1, NM_013982.1, NM_013983.1, NM_013984.1, NM_013985.1) | Hs00171706_m1 | 5q23-q33 |
| ***** | NPM2 (NM_182795.1) | Hs00402011_m1 | 8p21.3 |
| ***** | EGF (NM_001963.2) | Hs00153181_m1 | 4q25 |
| ***** | NCOA1 (NM_147223.2,NM_147233.2,NM_003743.4) | Hs00186661_m1 | 2p23 |
| ***** | PGR (NM_000926.2) | Hs00172183_m1 | 11q22-q23 |
| ***** | PRKCE (NM_005400.2) | Hs00178455_m1 | 2p21 |
|  | PRKCA (NM_002737.2) | Hs00176973_m1 | 17q22-q23.2 |
| ***** | RARA (NM_000964.1) | Hs00230907_m1 | 17q21 |
| ***** | XBP1 (NM_005080.2) | Hs00231936_m1 | 22q12.1 |
| ***** | NAT1 (BC013732) | Hs00377717_m1 | 8p23.1-p21.3 |
| **¥** | RPLP0 (NM_053275.3,NM_001002.3) | Hs99999902_m1 | 12q24.2 |
|  | PRKAR1A (NM_212471.1,NM_212472.1,NM_002734.3) | Hs00267597_m1 | 17q23-q24 |
|  | IGF2 (NM_000612.2) | Hs00171254_m1 | 11p15.5 |
|  | CTSD (NM_001909.3) | Hs00157205_m1 | 11p15.5 |
|  | PLAUR (NM_001005376.1,NM_001005377.1,NM_002659.2) | Hs00182181_m1 | 19q13 |
| ***** | ESR2 (NM_001437.1) | Hs00230957_m1 | 14q23.2 |
| ***** | ERBB4 (NM_005235.1) | Hs00171783_m1 | 2q33.3-q34 |
| ***** | TNF (NM_000594.2) | Hs00174128_m1 | 6p21.3 |
|  | BCAR1 (NM_014567.2) | Hs00183953_m1 | 16q22-q23 |
| ***** | ESR1 (NM_000125.2) | Hs00174860_m1 | 6q25.1 |
|  | PLAU (NM_002658.2) | Hs00170182_m1 | 10q24 |
| ***** | HOXB13 (NM_006361.4) | Hs00197189_m1 | 17q21.2 |
| ***** | CDKN1A (NM_078467.1,NM_000389.2) | Hs00355782_m1 | 6p21.2 |
|  | IRS1 (NM_005544.1) | Hs00178563_m1 | 2q36 |
| ***** | IL17BR (NM_018725.2,NM_172234.1) | Hs00218889_m1 | 3p21.1 |
|  | BCAR3 (NM_003567.2) | Hs00182488_m1 | 1p22.1 |
| ***** | AKT1 (NM_001014431.1,NM_001014432.1,NM_005163.2) | Hs00178289_m1 | 14q32.32 |
| ***** | IRF1 (NM_002198.1) | Hs00233698_m1 | 5q31.1 |
|  | STS (NM_000351.3) | Hs00165853_m1 | Xp22.32 |
|  | AREG (NM_001657.2) | Hs00155832_m1 | 4q13-q21 |
|  | CCNE1 (NM_001238.1,NM_057182.1) | Hs00233356_m1 | 19q12 |
|  | TGFB1 (NM_000660.3) | Hs99999918_m1 | 19q13.2-.1 |
| ***** | AKT2 (NM_001626.2) | Hs00609846_m1 | 19q13.1-.2 |
| ***** | TFF1 (NM_003225.2) | Hs00170216_m1 | 21q22.3 |
| ***** | ERBB3 (NM_001005915.1,NM_001982.2) | Hs00176538_m1 | 12q13 |
|  | TNFRSF1A (NM_001065.2) | Hs01042313_m1 | 12p13.2 |
| ***** | ERBB2 (NM_001005862.1,NM_004448.2) | Hs00170433_m1 | 17q21.1 |
| ***** | BCL2 (NM_000633.2) | Hs00608023_m1 | 18q21.33 |
|  | BCAS3 (NM_017679.2) | Hs00375126_m1 | 17q23 |
|  | DUSP6 (NM_001946.2) | Hs00169257_m1 | 12q22-q23 |
