# Supplementary material for: Gene Expression Signatures That Predict Outcome of Tamoxifen-Treated Estrogen Receptor-Positive, High-Risk, Primary Breast Cancer Patients: A DBCG Study
Source: PLoS One. 2013 Jan 16;8(1):e54078. doi: 10.1371/journal.pone.0054078 (PMC3546921; doi:10.1371/journal.pone.0054078)
Supplement: Supplementary Material S4 — Identified Genes and corresponding probes in previously-published microarray datasets. (DOC) [file pone.0054078.s004.doc]

**Supplementary material S4**:

A complete list of the genes identified by real-time PCR and the presence of corresponding probes in previously-published microarray datasets. Since the 9-gene signature was identified by using the 59 initially analyzed genes, two of these nine genes (*CGA* and *BCAR3*) were not part of the reduced gene set of 27 genes. The platform GSE6532-GPL97 was excluded due to the unavailability of several genes (grey text). NA: probe for the gene was not available. +: at least one probe for the gene was available.

| **Genes** | **Profiles (genes)** | | | **Microarray studies** | | | | | | |
| --- | --- | --- | --- | --- | --- | --- | --- | --- | --- | --- |
|  | **2** | **8** | **9** | **6532-gpl96** | **6532-gpl97** | **6532-gpl570** | **1378** | **1379** | **9893** | **12093** |
| *AKT2* | - | - | - | + | + | + | + | + | + | + |
| *BCL2* | x | x | x | + | NA | + | + | + | + | + |
| *CDKN1A* | x | - | x | + | NA | + | + | + | + | + |
| *EGF* | - | - | - | + | NA | + | + | + | + | + |
| *ESR1* | - | x | - | + | NA | + | + | + | + | + |
| *IGF1R* | - | x | - | + | + | + | + | + | + | + |
| *IL17BR* | - | - | - | + | + | + | + | + | + | + |
| *IRF1* | - | - | - | + | + | + | + | + | + | + |
| *NAT1* | - | - | x | + | NA | + | + | + | NA | + |
| *NCOA1* | - | x | - | + | + | + | + | + | + | + |
| *NPM2* | - | - | - | NA | + | + | NA | NA | NA | NA |
| *NRG1* | - | x | - | + | NA | + | + | + | + | + |
| *PRKCD* | - | x | - | + | NA | + | + | + | + | + |
| *PRKCE* | - | x | x | + | + | + | + | + | + | + |
| *RARA* | - | - | - | + | NA | + | + | + | + | + |
| *SERPINE1* | - | - | - | + | NA | + | + | + | + | + |
| *TNF* | - | - | - | + | NA | + | NA | NA | + | + |
| *XBP1* | - | - | - | + | NA | + | + | + | + | + |
| *EGFR* | - | x | x | + | NA | + | + | + | + | + |
| *AKT1* | - | - | x | + | NA | + | + | + | + | + |
| *TFF* | - | - | x | + | NA | + | + | + | + | + |
| *ERBB2* | - | - | - | + | + | + | + | + | + | + |
| *ERBB3* | - | - | - | + | + | + | + | + | + | + |
| *ERBB4* | - | - | - | + | + | + | + | + | + | + |
| *ESR2* | - | - | - | + | NA | + | + | + | + | + |
| *HOXB13* | - | - | - | + | + | + | + | + | + | + |
| *PGR* | - | - | - | + | + | + | + | + | + | + |
