# Supplementary material for: Gene Expression Signatures That Predict Outcome of Tamoxifen-Treated Estrogen Receptor-Positive, High-Risk, Primary Breast Cancer Patients: A DBCG Study
Source: PLoS One. 2013 Jan 16;8(1):e54078. doi: 10.1371/journal.pone.0054078 (PMC3546921; doi:10.1371/journal.pone.0054078)
Supplement: Supplementary Material S5 — Cox regression analysis of the independent datasets. (DOCX) [file pone.0054078.s005.docx]

**Supplementary material S5**

*Cox analysis of the independent datasets investigating the 2-gene signatures performance.* Abbreviations: RR: relative risk. CI: confidence interval.

A) Univariate Cox regression analysis and Kaplan-Meier plots of previously published gene expression datasets (microarray). The Kaplan-Meier plots are depicted as probability of survival vs. days. B) Table shows the multivariate analysis providing first the p-value, followed by the RR, and CI range.

A)

| **Dataset** | **p-value** | **RR** | **95% CI** |
| --- | --- | --- | --- |
| GSE1378 | 0.22 | 1.63 | 0.74 – 3.55 |
| GSE1379 | 0.0094 | 2.78 | 1.28 – 5.92 |
| GSE6532, GPL96 | 0.075 | 0.16 | 0.02 – 1.20 |
| GSE6532, GPL570 | <0.0001 | 10.77 | 4.92 – 23.57 |
| GSE9893 | 0.052 | 1.74 | 0.10 – 3.04 |
| GSE12093 | 0.43 | 1.47 | 0.56 – 3.85 |


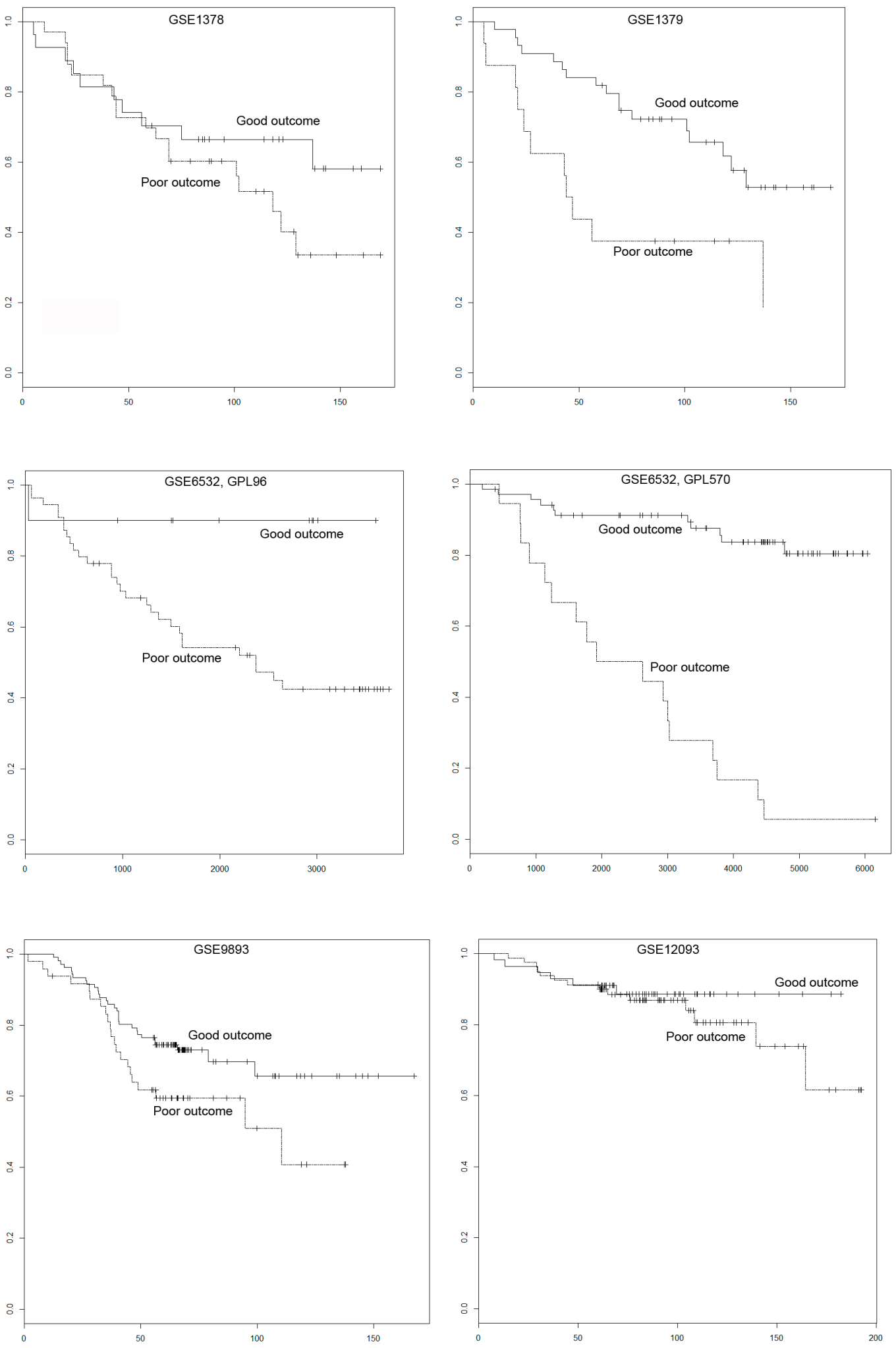


B)

|  | **GSE1378** | **GSE1379** | **GSE6532, GPL96** | **GSE6532, GPL570** | **GSE9893** | **GSE12093** |
| --- | --- | --- | --- | --- | --- | --- |
| 2-genes | 0.24 | 0.17 | 0.36 | <0.0001 *** | 0.16 | NA |
|  | 1.74 | 2.08 | 0.38 | 18.30 | 1.58 |  |
|  | (0.69-4.40) | (0.73-5.89) | (0.05-3.04) | (6.32-53.00) | (0.83-2.98) |  |
| Age | 0.28 | 0.72 | 0.66 | 0.37 | 0.66 | NA |
|  | 0.97 | 0.99 | 0.99 | 0.97 | 0.99 |  |
|  | (0.93-1.02) | (0.95-1.04) | (0.95-1.03) | (0.90-1.04) | (0.96-1.03) |  |
| Grade | 0.37 | 0.85 | 0.16 | 0.14 | 0.0006 *** | NA |
|  | 1.53 | 0.91 | 2.77 | 1.93 | 2.49 |  |
|  | (0.60-3.94) | (0.33-2.52) | (0.66-11.53) | (0.81-4.61) | (1.48-4.18) |  |
| Size | 0.82 | 0.55 | 0.91 | 0.032 * | 0.92 | NA |
|  | 1.05 | 1.23 | 1.03 | 1.58 | 1.00 |  |
|  | (0.69-1.60) | (0.78-1.68) | (0.63-1.69) | (1.04-2.41) | 0.96-1.05) |  |
| Lymph nodes | 0.18 | 0.18 | 0.087 | 0.025 * | 0.002 ** | NA |
|  | 1.05 | 1.05 | 2.33 | 0.28 | 3.08 |  |
|  | (0.10-1.12) | (0.98-1.12) | (0.88-6.13) | (0.09-0.85) | (1.51-6.28) |  |
